# Anterior and Posterior Syndesmotic Augmentation Using Nonabsorbable Suture Tape for Acute Syndesmotic Instability: A Technical Note

**DOI:** 10.3390/jcm14072207

**Published:** 2025-03-24

**Authors:** Si-Wook Lee, Sung-Joon Yoon, Ki-Jin Jung, Eui-Dong Yeo, Sung-Hun Won, Chang-Hwa Hong, Soon-Do Wang, Yong-Chan Cho, Jae-Young Ji, Je-Yeon Byeon, Dhong-Won Lee, Woo-Jong Kim

**Affiliations:** 1 Department of Orthopedic Surgery, Dongsan Medical Center, Keimyung University, Daegu 42601, Republic of Korea; shuk2000@naver.com; 2 Department of Orthopaedic Surgery, Soonchunhyang University Hospital Cheonan, Cheonan 31151, Republic of Korea; yunsj0103@naver.com (S.-J.Y.); c89546@schmc.ac.kr (K.-J.J.); chhong@schmc.ac.kr (C.-H.H.); gether@schmc.ac.kr (S.-D.W.); dhsdyd12@naver.com (Y.-C.C.); 3 Department of Orthopaedic Surgery, Veterans Health Service Medical Center, Seoul 05368, Republic of Korea; angel_doctor@naver.com; 4 Department of Orthopaedic Surgery, Soonchunhyang University Hospital Seoul, 59, Daesagwan-ro, Yongsan-gu, Seoul 04401, Republic of Korea; orthowon@schmc.ac.kr; 5 Department of Anesthesiology and Pain Medicine, Soonchunhyang University Hospital Cheonan, 31, Suncheonhyang 6-gil, Dongam-gu, Cheonan 31151, Republic of Korea; phmjjy@naver.com; 6 Department of Plastic Surgery, Soonchunhyang University Hospital Cheonan, 31, Suncheonhyang 6-gil, Dongam-gu, Cheonan 31151, Republic of Korea; 115954@schmc.ac.kr; 7 Department of Orthopaedic Surgery, Konkuk University Medical Center, 120-1, Neungdong-ro, Gwangjin-gu, Seoul 05030, Republic of Korea; wonbayo@naver.com

**Keywords:** syndesmosis, posterior inferior tibiofibular ligament, syndesmotic instability, suture tape, augmentation

## Abstract

**Background:** Syndesmotic instability presents a significant challenge in orthopedic surgery, with no universally accepted treatment approach. Traditional methods, such as trans-syndesmotic screw fixation, remain widely used but are associated with complications, including malreduction, hardware-related issues, and restricted physiological motion. Recent advancements in flexible dynamic fixation, particularly suture tape augmentation, offer promising alternatives by preserving native biomechanics and enabling early rehabilitation. **Methods:** This technical note details an anterior-to-posterior syndesmotic augmentation technique using suture tape to address unstable syndesmotic injuries involving both the anterior inferior tibiofibular ligament and posterior inferior tibiofibular ligament. The proposed technique aims to restore stability, reduce complications, and improve functional outcomes. **Results:** The technique eliminates the need for hardware removal, supports early weight-bearing, and minimizes postoperative limitations. **Conclusions:** Anterior-to-posterior syndesmotic augmentation with suture tape represents a viable alternative for syndesmotic instability. Further clinical and biomechanical studies are warranted to validate its long-term efficacy and applicability across diverse patient populations.

## 1. Introduction

The ankle joint, often referred to as the body’s largest “mortise” joint, ensures stability and mobility by accommodating the talus within the mortise formed by the distal tibia and fibula through the syndesmosis [1]. This syndesmosis serves as a true articulation, reinforced by the tibiofibular ligaments. These ligaments maintain the stability between the tibia and fibula by resisting axial, rotational, and translational forces, while also preventing diastasis and limiting external rotation of the fibula [2,3,4]. Structurally, the syndesmosis comprises the following three primary ligaments: the anterior inferior tibiofibular ligament (AITFL), the interosseous ligament, and the posterior inferior tibiofibular ligament (PITFL) [3].

Ankle fractures are among the most prevalent lower extremity injuries, with reports indicating that approximately 20–45% of these cases are associated with distal syndesmosis injuries [5,6]. Various approaches have been developed for managing ankle fractures with syndesmosis injuries. Conservative strategies, such as protected weight-bearing using a cast or boot for a specified duration, are commonly used. Surgically, trans-syndesmotic screw fixation (static fixation) has been a cornerstone technique for many years [7,8]. However, limitations, such as syndesmosis malreduction and screw breakage, have prompted the development of newer fixation methods that aim to maintain stability while allowing native ankle and distal tibiofibular joint motion [9,10].

Flexible dynamic fixation using suture buttons has addressed some of the shortcomings of screw fixation [11,12,13,14]. Nevertheless, concerns about the potential lack of stability with these constructs, particularly in rotational and sagittal plane motions, persist. To address these challenges, reinforcement techniques involving suture tape have been introduced and demonstrated improved stability [15,16,17,18]. Additionally, previous studies have demonstrated that, in physiological environments, nonabsorbable sutures tend to maintain their mechanical strength, which suggests that braided suture tape could provide superior stability compared to traditional fixation methods [19,20]. Furthermore, surgical techniques using suture tape alone have shown promising results in several studies [21,22,23,24,25]. A comparative analysis of the characteristics of suture tape augmentation and suture button fixation is summarized in Table 1.

Despite these advancements, most fixation techniques primarily focus on injuries involving the AITFL, with limited attention to more severe Grade III injuries [2], including intraoperative Grade 2 and 3 injuries [26,27], which involve PITFL disruption. Recognizing this gap, we propose a surgical technique that uses suture tape to address PITFL injuries specifically. This approach aims to enhance stability while preserving the native biomechanics of the joint. This study sought to contribute to the management of syndesmotic injuries by addressing these unmet clinical needs.

## 2. Preoperative Assessment and Imaging

Thorough preoperative evaluation is critical to determine the fracture pattern and assess variations in syndesmotic alignment. This includes assessing the tibiofibular clear space and tibiofibular overlap using standard X-rays in anteroposterior, lateral, and mortise views, as well as obtaining full-length radiographs of the lower leg to evaluate the possibility of high fibula fractures. Achieving accurate fracture reduction is a prerequisite before proceeding with syndesmosis augmentation. All necessary fracture reductions and fixations must be completed prior to initiating the syndesmosis augmentation procedure.

Magnetic resonance imaging (MRI), when available, provides valuable information regarding deltoid ligament ruptures, syndesmotic injuries, or intra-articular abnormalities. In addition, the authors routinely perform arthroscopy both before and after syndesmosis augmentation. This allows for the evaluation of the syndesmotic reduction accuracy and facilitates the management of any intra-articular pathological lesions [28,29,30].

## 3. Surgical Technique

This technical note was approved by the Institutional Review Board (IRB) of Soonchunhyang University Cheonan Hospital, Cheonan, South Korea (IRB No. 2025-01-021). Written informed consent for the publication of accompanying images was obtained from the patient.

The procedure was conducted under general anesthesia or lower extremity nerve blocks, with the patient in the supine position. The lower extremity was prepared and draped in a standard sterile fashion, and a tourniquet was applied and inflated to maintain a bloodless surgical field. An elastic bandage was used to retract the ankle joint. An anteromedial portal and an anterolateral portal were created for arthroscopic exploration. During arthroscopy, a 2-mm probe tip was passed through the widened tibiofibular joint to confirm the presence of a syndesmosis injury. Direct visualization also identified ruptures of both the AITFL and PITFL.

AITFL injuries were identified approximately 4 cm proximal to the distal tibiofibular joint through an anterolateral approach to the lateral malleolus [27,28]. Syndesmosis augmentation was performed using one 3.5-mm BioComposite™ SwiveLock^®^ anchor and two 4.75-mm BioComposite™ SwiveLock^®^ anchors (Arthrex, Naples, FL, USA), along with the associated drills and taps included in the InternalBrace™ Ligament Augmentation Repair system.

**Figure 1:**
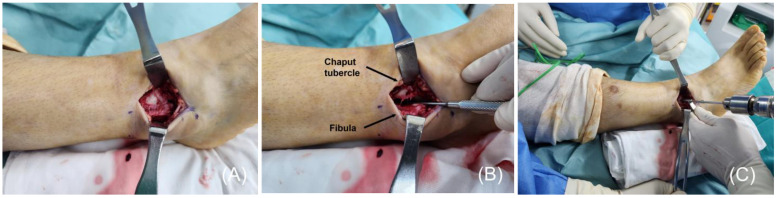
(**A**) The injury to the anterior inferior tibiofibular ligament (AITFL) is identified via an anterolateral approach. This step is occasionally performed in conjunction with the management of lateral malleolar fractures. (**B**) Complete rupture of the AITFL is confirmed by widening the tibiofibular joint using a Freer elevator. (**C**) The distal tibial footprints of the AITFL are visualized, and a 3.4-mm bone tunnel is created at the Chaput tubercle to secure the suture tape using a SwiveLock^®^ anchor.

The footprint of the AITFL on the Chaput tubercle of the distal tibia was identified, and a 3.4-mm bone tunnel was created. Depending on the surgeon’s preference, this step may be performed after completing PITFL augmentation (Figure 1). To address the PITFL injury, the patient’s leg was internally rotated, or the position was adjusted to lateral. A 5-cm posterolateral incision was made between the lateral malleolus and the Achilles tendon to expose the posterior aspect of the fibula and Volkmann tubercle. A 2.7-mm drill was used to create a bone tunnel through the fibula at the AITFL footprint, drilled from anterior to posterior.

**Figure 2:**
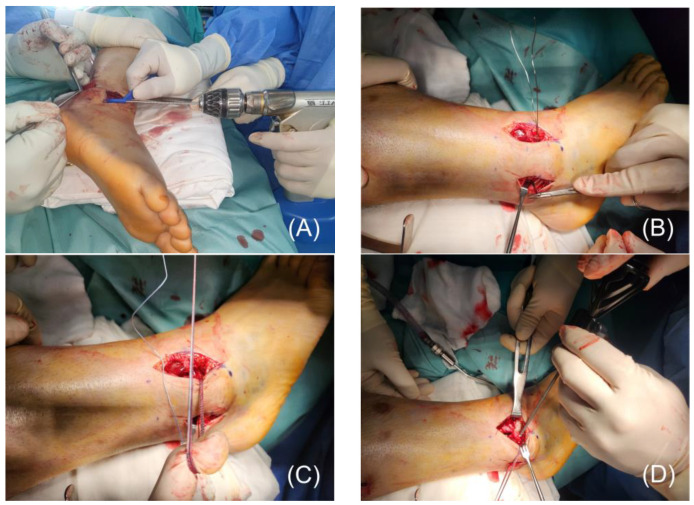
(**A**) A 2.7-mm drill is used to create a bone tunnel through the fibula at the AITFL footprint, drilled from anterior to posterior. (**B**) A thin, pre-twisted wire is inserted from posterior to anterior through the tunnel to facilitate the passage of the suture tape. (**C**) The suture tape is passed through the bone tunnel. (**D**) Fixation of the suture tape is achieved by securing it with a 3.5-mm SwiveLock^®^ interference screw.

To guide the suture tape through the bone tunnel, a thin, pre-twisted wire was initially inserted from posterior to anterior. The suture tape was securely attached to the wire’s distal end, which was then carefully retracted back through the tunnel. Once positioned, the suture tape was fixed using a 3.5-mm SwiveLock^®^ anchor as an interference screw (Figure 2).

**Figure 3:**
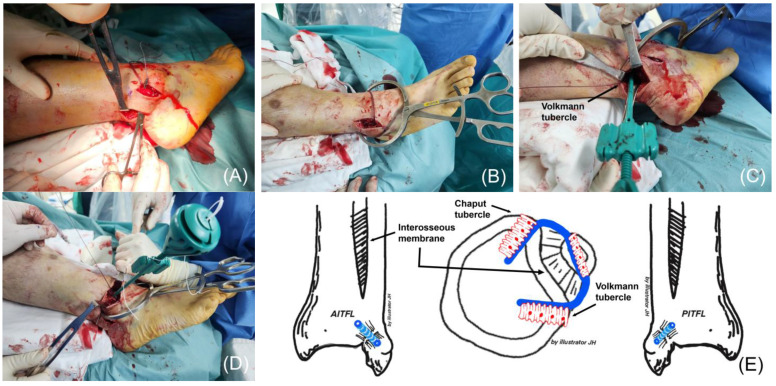
(**A**) The posterior suture tape is passed between the peroneus tendon and the bone. (**B**) Syndesmosis reduction is performed under direct visualization using a reduction clamp. (**C**) The posterior free end of the suture tape is secured in a pre-drilled tunnel at the Volkmann tubercle using a 4.75-mm SwiveLock^®^ anchor. (**D**) The anterior suture tape is then fixed to the Chaput tubercle, completing the syndesmosis augmentation. (**E**) Comprehensive illustration of the finalized compressive suture tape syndesmosis augmentation.

The posterior suture tape was passed between the peroneus tendon and the bone after identifying the Volkmann tubercle. The tape’s free end was secured to the tubercle through a 3.4-mm bone tunnel using a 4.75-mm SwiveLock^®^ anchor while maintaining syndesmotic reduction. Reduction was achieved with a reduction clamp, and temporary fixation was performed with a Kirschner wire (K-wire), if required. After securing the posterior side, the anterior suture tape was anchored to the Chaput tubercle with a 4.75-mm SwiveLock^®^ anchor, stabilizing the syndesmosis (Figure 3).

To ensure accurate placement and optimal fixation, meticulous attention must be paid during the procedure to prevent technical errors and associated complications. Potential complications include the overtightening or malpositioning of the suture tape, which may lead to excessive compression, syndesmotic malalignment, or the restriction of physiological movement. Additionally, improper drill placement or excessive tension during fixation may increase the risk of fibular fracture.

The reduction of the syndesmosis was then reassessed arthroscopically to ensure proper alignment, and the procedure was concluded.

Postoperatively, the patient was immobilized with a short leg splint for approximately 2 weeks, followed by an ankle brace for an additional 2 weeks, during which non-weight-bearing ambulation with crutches was recommended. Active and passive ankle range of motion (ROM) exercises were initiated at 4 weeks postoperatively, aiming to achieve at least 10° of dorsiflexion and 30° of plantar flexion by the end of the first month. Full weight-bearing walking with the brace was permitted, and the brace was removed at 6 weeks postoperatively.

A structured 12-week rehabilitation program was implemented, consisting of progressive strength training, balance exercises, and functional recovery protocols. During the first six weeks, the focus was on protecting the surgical site while allowing early mobilization. In the initial four weeks, patients were instructed to limit weight-bearing while wearing a brace, and gentle passive and active ROM exercises were introduced to prevent stiffness. By the end of six weeks, the target ROM was 15° dorsiflexion and 35° plantar flexion.

From weeks 7 to 12, the rehabilitation protocol emphasized strength and stability training. Patients were allowed to walk without a brace, and progressive resistance exercises were incorporated to strengthen the ankle dorsiflexors, plantarflexors, and stabilizing muscles. Additionally, balance training exercises, such as single-leg stance and proprioceptive drills, were introduced to improve postural stability. The ROM goal for this period was full dorsiflexion of 20° and full plantar flexion of 40°.

After week 12, the rehabilitation plan focused on functional recovery and return to pre-injury activities. For individuals engaged in high-demand physical activities, sport-specific training was introduced, and, if necessary, plyometric and agility exercises were added to restore dynamic movement control. Full return to daily activities was expected within 3 to 4 months postoperatively.

To objectively assess rehabilitation progress, outcome measures, including the Olerud–Molander Ankle Score (OMAS) and visual analog scale (VAS) for pain, were recorded at 3 months, 6 months, and 1 year postoperatively. These assessments were used to monitor functional recovery and patient-reported outcomes over time.

Follow-up axial computed tomography (CT) scans were performed at 6 months, 1 year, and annually thereafter to monitor healing and alignment.

The patient demographics and clinical analysis results are presented in Table 2.

## 4. Discussion

The PITFL is a robust syndesmotic ligament with the following two distinct components: the superficial PITFL and the deep PITFL [31]. Biomechanical studies have shown that PITFL rupture leads to syndesmotic instability [15,32,33]. Clanton et al. [15] demonstrated that the posterior inferior tibiofibular ligament (PITFL) plays a crucial role in syndesmotic stability, particularly in controlling internal rotation and sagittal plane translation of the fibula. Their biomechanical study revealed that internal rotational resistance was not significantly reduced until both the deep and superficial PITFL were severed, highlighting its stabilizing function. Additionally, they found that sectioning the superficial PITFL led to increased sagittal translation, indicating its role in preventing excessive anterior–posterior fibular movement. The deep PITFL provides primary structural support, while the superficial PITFL offers additional reinforcement, especially under rotational and axial loads. While isolated PITFL injury does not cause severe instability, its role becomes more significant when combined with AITFL or interosseous membrane injuries, leading to increased syndesmotic instability. Ogilvie-Harris et al. [33] demonstrated that syndesmosis stability is primarily maintained by the AITFL and the deep PITFL, contributing 35.5% and 32.7% to resistance against diastasis, respectively. The interosseous ligament accounts for 21.6% of the stability, while the superficial PITFL contributes 8.7%. Sequential sectioning of these ligaments progressively decreases stability, culminating in complete tibiofibular separation when all of the ligaments are severed. Disruption of two or more major ligaments reduces stability by over 50%, significantly increasing the risk of syndesmotic instability. Accurate assessment and appropriate treatment of the AITFL and deep PITFL are, therefore, essential to prevent chronic instability and associated complications. Similar findings have been reported in studies using arthroscopic probing techniques [32].

Various classification systems have been proposed to categorize syndesmotic injuries, with a simple grading system based on clinical symptoms and radiographic findings being the most widely used [34,35,36]. Grade I injuries involve stable syndesmosis with mild symptoms and normal radiographs, indicative of a ligamentous sprain that is managed conservatively. Grade II injuries are characterized by the partial disruption of the syndesmotic complex with variable instability. While radiographs may appear normal, positive provocative tests or MRI findings, such as concomitant deltoid ligament disruption, may imply the need for surgical stabilization [35]. Grade III injuries, involving complete disruption of the anterior and posterior syndesmotic complex with evident radiographic malalignment, necessitate surgical intervention [35,36].

Markus et al. [26,27] proposed an intraoperative grading system under direct visualization, categorizing injuries into three grades based on ligament involvement and instability. Grade 1 injuries involve isolated AITFL tears or bony avulsion, with increased external rotation and posterior sagittal translation. Grade 2 injuries include additional PITFL involvement, resulting in increased anterior sagittal translation. Grade 3 injuries exhibit medial instability with increased lateral translation. These findings emphasize the importance of stabilizing not only the AITFL but also the PITFL in Grade II and III injuries, forming the rationale for the surgical technique described in this study.

Suture tape has been increasingly used for flexible dynamic stabilization in ligament repairs. FiberTape^®^ (Arthrex^®^, Naples, FL, USA), a braided suture tape composed of ultra-high-molecular-weight polyethylene and polyester, offers an ultimate tensile strength of approximately 750 N [37]. The InternalBrace™ technique, introduced by Gordon Mackay in 2012, uses SwiveLocks^®^ (Arthrex^®^, Naples, FL, USA) for the knotless fixation of FiberTape^®^ at the anatomical ligament footprints [26]. This technique prioritizes ligament healing over replacement, leveraging the large original ligament footprints, which are challenging to replicate with tendon grafts [37].

A key advantage of the InternalBrace™ is the preservation of proprioception through the retention of ligament remnants. Acting as a stabilizing “seatbelt”, it promotes the early mobilization of repaired ligaments while supporting gradual tissue strengthening over time [37,38]. From the perspective that ideal syndesmotic fixation should allow for physiological movement, provide strong fixation, and enable early postoperative weight-bearing, suture tape augmentation has garnered significant interest [27,39].

Nelson introduced a direct anatomical repair technique for AITFL injuries in transmalleolar fractures, highlighting its ability to stabilize the ankle mortise and enhance bone healing. This approach obviates the need for syndesmotic screw fixation, facilitating faster recovery and earlier return to functional activities [40]. Lee et al. [41] expanded upon Nelson’s approach by developing an arthroscopic augmentation technique using suture tape. This minimally invasive method enables early weight-bearing and rehabilitation, eliminates the need for screw removal, and avoids postoperative functional limitations, offering an efficient and less-invasive alternative [40,41].

The application of suture tape in syndesmotic repair or augmentation has been the focus of several studies, consistently demonstrating favorable biomechanical and clinical outcomes. Lee et al. [23] conducted a biomechanical study using 10 matched pairs of human cadaver specimens to compare syndesmotic screw fixation with suture tape augmentation in treating syndesmotic injuries. Through cyclic loading and failure testing, they evaluated the torque-to-failure and angle-to-failure, and found no significant differences in the biomechanical stability between the two techniques. The authors concluded that open anterior syndesmotic repair augmented with suture tape offers torsional strength comparable to screw fixation, making it a viable alternative. Furthermore, its ability to preserve physiological motion and reduce the need for secondary surgeries enhances its clinical appeal.

Kwon et al. [25] evaluated nonabsorbable suture tape for AITFL augmentation in 66 patients with 68 syndesmotic injuries and reported effective stabilization with early weight-bearing at 3.3 weeks and unrestricted activity at 22.7 weeks. Complications were minimal (4.4%), with no cases of malalignment or diastasis. The study concluded that suture tape augmentation is a safe and effective technique for syndesmotic repair, allowing for expedited rehabilitation and avoiding complications associated with tendon grafts.

Jamieson et al. [22] investigated the effectiveness of AITFL suture repair and suture tape augmentation in restoring stability and kinematics in isolated syndesmotic injuries. Using 12 cadaveric lower leg specimens, they compared different repair techniques under simulated physiological and stress loads. Their results demonstrate that AITFL suture repair with suture tape augmentation most closely restored stability and the anatomical relationships of the tibia, fibula, and talus. While suture button fixation provided adequate stabilization, it tended to overcompress and externally rotate the fibula. Consequently, suture tape augmentation was deemed a valuable alternative, particularly for isolated syndesmotic injuries.

Harris et al. [24] examined suture tape augmentation for AITFL reconstruction in 19 elite athletes with unstable syndesmotic injuries. After a follow-up period of 17–35 months, all of the athletes returned to their pre-injury activity levels. For isolated injuries, the mean return-to-play time was 62 days, while concomitant injuries required an average of 104 days. Postoperative outcomes included a mean AOFAS score of 100, with knee-to-wall measurements comparable to the contralateral side in 18 patients. The authors concluded that suture tape augmentation offers effective stabilization, facilitating the early return to sports with excellent short-term outcomes.

While these studies predominantly focused on AITFL augmentation, the described suture tape techniques have a critical limitation: they are applicable only when the PITFL remains intact. To date, no studies specifically addressing PITFL augmentation or repair have been identified, representing a significant gap in the literature. Addressing this gap could expand the applicability of suture tape techniques to a broader range of syndesmotic injuries.

For suture tape techniques to achieve broader acceptance in clinical practice, several aspects require further investigation. First, larger patient cohorts must be studied to provide robust clinical data. Second, the long-term efficacy of suture tape augmentation needs to be established through extended follow-up studies. Third, multicenter studies are necessary to enhance the generalizability of the findings across diverse populations and healthcare systems.

Elghazy et al. [11] compared screw and suture button fixation for syndesmosis instability using weight-bearing CT scans, reporting that neither method fully restored the syndesmotic area compared to the contralateral uninjured ankle. The suture button group exhibited external rotation of the fibula, contributing to increased asymmetry in anterior and direct anterior measurements. Considering the biomechanical advantages of suture tape, future studies should employ weight-bearing CT scans to evaluate its efficacy in syndesmotic fixation. Moreover, cadaveric biomechanical studies should focus on concurrent AITFL and PITFL injuries, comparing the following three groups: screw fixation, suture button fixation with AITFL suture tape augmentation, and combined AITFL and PITFL suture tape augmentation. These investigations would provide critical insights into the relative effectiveness of these techniques in restoring stability and preserving joint biomechanics, thereby validating the role of suture tape in the comprehensive management of syndesmotic injuries.

## 5. Conclusions

Anterior-to-posterior syndesmotic augmentation with suture tape presents a promising alternative for managing unstable syndesmotic injuries. By addressing injuries to both the AITFL and PITFL, this technique preserves native joint biomechanics, facilitates early rehabilitation, and eliminates the need for hardware removal.

Despite its potential to improve clinical outcomes, this study has several limitations. First, the lack of long-term clinical follow-up data limits our ability to assess the durability and effectiveness of this technique over time. Second, the absence of large-scale comparative studies makes it difficult to establish its superiority over conventional fixation methods. Third, this study does not include cadaveric biomechanical validation, which would provide further insight into the technique’s mechanical stability and functional outcomes.

Further validation through large-scale clinical trials and long-term follow-up studies is essential. Future research should focus on comparing this technique with traditional fixation methods under diverse biomechanical conditions and across broader patient demographics. Additionally, cadaveric studies that investigate combined AITFL and PITFL injuries would provide valuable insights into the biomechanical efficacy of this approach, supporting its optimization and wider adoption in clinical practice.

## Figures and Tables

**Table 1:** Comparison of suture tape augmentation and suture button fixation.

Feature	Suture Tape Augmentation	Suture Button Fixation
Stability	Provides strong initial stabilization and support for ligament healing.	Offers dynamic stabilization with controlled micromotion, allowing for some physiological movement.
Biomechanical Strength	Reinforces ligament structures and helps restore anatomical alignment.	More flexible than screws and maintains stability without excessive stiffness.
Risk of Implant Failure	Lower risk of breakage compared to screws, but potential loosening over time.	Higher risk of button migration or malreduction if improperly placed.
Flexibility in Load Transfer	Distributes forces evenly across the repaired ligament structures.	Allows for partial mobility, which may help prevent stress-shielding effects.
Postoperative Rehabilitation	Earlier weight-bearing compared to screws; reduced need for implant removal.	Allows for early mobilization but may require additional stabilization if used alone.
Clinical Outcomes	Generally good functional outcomes, especially in high-activity individuals.	Some studies suggest better outcomes in preserving syndesmotic motion.
Complications	Risk of ligament stretching over time, requiring careful postoperative monitoring.	Potential for button prominence and irritation.

**Table 2:** Patient demographics and results.

Pt. No.	Age	Sex	Cause	Lauge–Hansen Classification	Injured Side	Follow-Up (mo)	OMAS		VAS Score		Complications
							Pre	Post	Pre	Post	
1	23	M	S	SER IV	Left	9	10	80	8	1	None
2	46	M	S	PER IV	Right	8	0	85	7	0	None
3	36	M	S	PER IV	Right	16	0	90	8	0	None
4	37	F	S	PER IV	Left	12	0	95	8	0	None
5	46	M	S	SER IV	Left	9	0	80	9	1	None
6	39	M	TA	PER IV	Right	12	0	90	8	0	None
7	19	F	S	SER IV	Left	9	0	85	9	0	None
Mean	35.14	NA	NA	NA	NA	10.71	1.43	85.71	8.00	0.29	NA
SD	10.51	NA	NA	NA	NA	2.81	3.78	5.35	0.82	0.49	NA
*p* value								<0.001		<0.001	

Abbreviations: Pt. No., patient number; OMAS, Olerud–Molander Ankle Score; VAS, visual analog scale; Pre, preoperative; Post, postoperative; F, female; M, male; S, slip down; TA, traffic accident; SER, supination external rotation; PER, pronation external rotation NA, not applicable; SD, standard deviation.

## Data Availability

Data sharing is not applicable to this article as no datasets were generated or analyzed during the current study.

## Abbreviations

AITFL	anterior inferior tibiofibular ligament
PITFL	posterior inferior tibiofibular ligament
MRI	magnetic resonance imaging
CT	computed tomography
ROM	ankle range of motion
OMAS	Olerud–Molander ankle score
VAS	visual analog scale
